# Decoding Individual Finger Movements from One Hand Using Human EEG Signals

**DOI:** 10.1371/journal.pone.0085192

**Published:** 2014-01-08

**Authors:** Ke Liao, Ran Xiao, Jania Gonzalez, Lei Ding

**Affiliations:** 1 School of Electrical and Computer Engineering, University of Oklahoma, Norman, Oklahoma, United States of America; 2 Center for Biomedical Engineering, University of Oklahoma, Norman, Oklahoma, United States of America; University of Maryland, College Park, United States of America

## Abstract

Brain computer interface (BCI) is an assistive technology, which decodes neurophysiological signals generated by the human brain and translates them into control signals to control external devices, e.g., wheelchairs. One problem challenging noninvasive BCI technologies is the limited control dimensions from decoding movements of, mainly, large body parts, e.g., upper and lower limbs. It has been reported that complicated dexterous functions, i.e., finger movements, can be decoded in electrocorticography (ECoG) signals, while it remains unclear whether noninvasive electroencephalography (EEG) signals also have sufficient information to decode the same type of movements. Phenomena of broadband power increase and low-frequency-band power decrease were observed in EEG in the present study, when EEG power spectra were decomposed by a principal component analysis (PCA). These movement-related spectral structures and their changes caused by finger movements in EEG are consistent with observations in previous ECoG study, as well as the results from ECoG data in the present study. The average decoding accuracy of 77.11% over all subjects was obtained in classifying each pair of fingers from one hand using movement-related spectral changes as features to be decoded using a support vector machine (SVM) classifier. The average decoding accuracy in three epilepsy patients using ECoG data was 91.28% with the similarly obtained features and same classifier. Both decoding accuracies of EEG and ECoG are significantly higher than the empirical guessing level (51.26%) in all subjects (*p*<*0.05*). The present study suggests the similar movement-related spectral changes in EEG as in ECoG, and demonstrates the feasibility of discriminating finger movements from one hand using EEG. These findings are promising to facilitate the development of BCIs with rich control signals using noninvasive technologies.

## Introduction

Brain-computer interface (BCI) technologies [1], [2], [3], [4] decode signals from brain activities and translate human intentions into commands to control external devices or computer applications. They provide alternative channels for people suffering from severe motor disabilities to perform necessary motor functions in daily life, bypassing damaged peripheral nerves and muscles.

Various brain signals have been adopted in BCI, including electroencephalography (EEG) [5], [6], electrocorticography (ECoG) [7], [8], electromyography (EMG) [9], [10], functional magnetic resonance imaging (fMRI) [11], [12], magnetoencephalography (MEG) [13], and near-infrared spectroscopy (NIRS) [14]. Among them, ECoG and EEG signals are two widely used modalities in BCI since they both reflect the electrical responses of the human brain in actions and their recording devices are more portable than others. ECoG records neuroelectrical signals of the brain with high quality and spatial resolution, which allow rapid user training and fast communication rates in BCI [15]. Many studies have been carried out using ECoG to extract control signals for BCI [16], [17]. However, ECoG is limited due to its invasiveness, which requires clinical surgery to place electrodes on the surface of the human brain. On the contrary, EEG records signals generated by same neuroelectrical activities on the scalp and its noninvasiveness makes it more practically usable than ECoG in BCI.

Different patterns in brain signals discussed above due to activations of different functional brain regions have been identified and extracted as control features for BCI, such as event-related synchronization and de-synchronization (ERS/ERD) originated from the motor cortex during real movement or motor imagery of certain body parts [6], [7], P300 component in evoked potentials from the parietal lobe [18], steady-state visually evoked potentials (SSVEP) from the occipital lobe [19], etc. In comparison to other features, control features related to motor functions are able to provide self-initiated stimulus-free control paradigm for BCI users, which fit better for applications involving movement controls. During the past decade, movements of large body parts have been investigated in EEG-based BCI, including wrists [20], upper limbs [21], elbows and shoulders [22], legs [23], and tongue [24]. However, the movements of fine body structures, such as individual fingers from one hand, have not been well studied in EEG-based BCI, while they are the most dexterous part of our body and play an irreplaceable role in our daily activities. For example, the flexion and extension of individual fingers are of great importance to compose many complicated movements. Some underlying shortcomings of EEG may account for this laggard. Firstly, EEG has a coarse spatial resolution with sensors around 10 millimeters apart [25]. Each sensor records EEG potentials from thousands of neurons or more [26], which are spatially filtered and superimposed. This limited spatial resolution imposes difficulties when using EEG to decode individual finger movements from one hand, which elicit close cortical motor areas. Secondly, the neuron populations on the motor cortex elicited by individual finger movements are smaller than those by large body parts [27]. Because EEG electrodes are placed outside of the scalp and relatively far from the brain compared to invasive technologies, recorded signals are greatly damped due to the volume conductor effect [28]. These facts indicate that EEG signals have limited signal-to-noise ratio (SNR) and bandwidth and it is thus a challenging task to decode fine dexterous movements [4], [29], [30], such as of individual fingers from one hand, using EEG [31]. Recently, EEG signals have been reported to decode different imaginary movements of wrists [20], directions of hand movements [32], the difference between wrist and finger movements [33], and even reconstruct three-dimensional (3D) hand movement paths [5]. All these studies have indicated that there is rich information in EEGs about fine dexterous movements. The difficulty is how movement-related information can be reliably extracted from EEG signals.

Recent ECoG-based BCI studies have shown promising results in extracting spatio-spectral features for individual finger movements [34]–[42]. Characteristic spectral changes at high frequency band (76–100 Hz) in ECoG have been reported during individual finger movements [34], which discriminate movements performed by thumb and index fingers in both contralateral and ipsilateral cases. It has been shown that time courses during finger flexion are highly correlated with ECoG data [35], [36] and can be reconstructed from ECoG data [37]. Individual finger movements have also successfully classified from ECoG [38]–[40] and micro-ECoG grid recordings [41]. Particularly, one recent ECoG study [42] suggests a broadband (up to 200 Hz) spectral power increase and characteristic spectral power decreases in both alpha (8–12 Hz) and beta (13–30 Hz) bands during individual finger movements from one hand, in which the broadband phenomenon has been demonstrated sensitive to movements performed by different fingers. These ongoing ECoG studies have demonstrated the feasibility of decoding individual finger movements using electrical potentials generated by the human brain, inspiring research in such decoding tasks using noninvasive EEG.

The objective of present study was to broaden the inventory of control signals for noninvasive BCIs via decoding individual finger movements from one hand using EEG. The power spectrum decoupling procedure [42] and principal components analysis method [43] were applied on EEG data acquired during individual finger movements to reveal the underlying movement-related spectral structure in EEG as compared to ECoG. The extracted features could extend various existing signals employed in state-of-the-art EEG-based BCI systems. Furthermore, the new features were applied to decode individual finger movements pairwise from one hand in order to validate their efficacy in the decoding task. The successful decoding of individual finger movements using EEG could facilitate developing noninvasive BCIs with more controls and complicated movement functions.

## Materials and Methods

### 1. Experimental protocol and data acquisition

Eleven healthy and right-handed subjects (1 female and 10 males, mean age: 26.4 years old, range: 22–32 years old) participated in this study given their written informed consents. The study was approved by the Institutional Review Board of the University of Oklahoma. None of these subjects had prior training on the experimental procedure in the present study. Due to poor data quality, data from one subject was excluded from further analysis.

**Figure 1:**
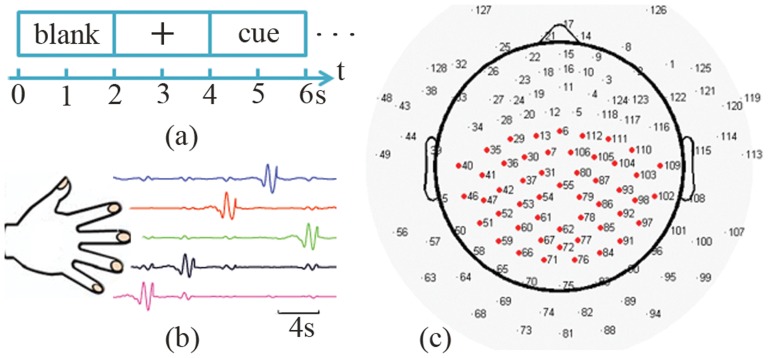
Experimental protocol and EEG sensor layout. (a) Events in each trial: a two-second blank window, a two-second fixation, and a two-second cue for finger movements. (b) Illustration of potentials difference during individual finger movements (no data in blank windows). (c) Illustration of a 128-channel EEG sensor layout with 50 electrodes (in red) as the mostly used channel set for decoding.

EEG experiments were carried out in a shielded chamber room. Subjects were seated in a comfortable armchair, with their arms supported in a supine position. They were instructed to perform flexion and extension of individual fingers according to visually presented cues in a LCD monitor using E-Prime software (Psychology Software Tools, Inc., Pittsburgh, PA, USA). During experiments, EEG signals were recorded from a 128-electrode EEG system (Geodesic EEG System 300, Electrical Geodesic Inc., OR, USA), sampled at either 250 Hz (in the first 6 subjects) or 1000 Hz (in the remaining 5 subjects) and referenced to the channel on the vertex. At the same time, the movements of individual fingers generated potential differences [44] (Fig. 1(b)), which were measured by five bipolar electrodes placed on both sides of each finger [44], [45] at the same sampling rate as in EEG. Real-time videos on the moving hands were recorded, for the purpose of removing trials from further analysis when subjects moved wrong fingers.

The experimental protocol was illustrated in Fig. 1(a). At the beginning of each trial, two-second blank window on the computer screen allowed subjects to prepare for the coming trial. Subjects were instructed to relax, blink, and swallow only in this period. A fixation cross was then presented for another two seconds as a resting condition, during which subjects were required to look at the fixation cross without movements. After that, one of the five words (i.e., thumb, index, middle, ring, little) as a cue was displayed for two seconds, which instructed subjects to continuously perform full flexion and extension of the cued finger (usually twice in one trial). There were in total 60 or 80 trials for each finger in all subjects (table 1).

**Table 1:** Numbers of EEG experimental trials, detected finger movements, and detected individual finger movements in all subjects.

	Trials	Movements	Thumb	Index	Middle	Ring	Little
Subject 1	400	409	93	79	80	78	79
Subject 2	400	485	87	97	117	97	87
Subject 3	300	380	74	59	84	81	82
Subject 4	400	435	83	77	86	80	109
Subject 5	400	396	68	105	88	71	64
Subject 6	400	396	80	79	80	79	78
Subject 7	400	394	79	77	80	79	79
Subject 8	400	394	80	80	79	75	80
Subject 9	400	395	80	80	75	80	80
Subject 10	400	394	80	79	80	75	80
Average	390	407.8	80.4	81.2	84.9	79.5	81.8

To evaluate extracted features from EEG and associated decoding performance, ECoG data from the BCI Competition IV [16], [46] was also analyzed for the purpose of comparison. The data were recorded from three epileptic patients using implanted 62-, 48- and 64-electrode grids, respectively, when they performed similar individual finger movements as in the present EEG study. Briefly, subjects were cued to move one of five fingers from the hand contralateral to implanted grids, with each cue lasting two seconds and followed by a two-second resting period. The visual cues were presented using BCI2000 [47]. During each cue, subjects typically moved the corresponding finger 3 to 5 times. ECoG signals were recorded for 10 minutes, digitized at 1000 Hz, and bandpass filtered (0.15–200 Hz). Kinematic data during finger movements were simultaneously recorded using a data glove (Fifth Dimension Technologies, Irvine, CA).

### 2. Data analysis

#### 2.1. Preprocessing

EEG data were high-pass filtered at 0.3 Hz using an elliptic infinite impulse response (IIR) filter from the EEGLAB toolbox [48] with both forward and reverse filtering to avoid phase distortions. Power line noise was removed by a 60 Hz notch filter with the transition band of 0.3 Hz. Independent component analysis (ICA) method [49] implemented in the EEGLAB toolbox based on the Infomax algorithm [50] was used to decompose EEG data into independent components (ICs). ICs related to common artifacts, such as generic discontinuities, electrooculogram (EOG), electrocardiogram (ECG), and electromyogram (EMG), were detected and rejected using the ADJUST toolbox [51]. Usually 10 to 20 ICs were rejected as artifacts in each subject.

For ECoG data, the 60 Hz power line noise and its harmonic components were removed using a notch filter with 0.8 Hz transition band (elliptic IIR filter from EEGLAB). Channels that contain unusually large values (greater than 10^5^ µV) were rejected as bad channels, resulting 61, 46 and 63 channels of ECoG data for each subject, respectively.

#### 2.2. Detection of finger movements

Since subjects usually performed finger movements twice in each trial, i.e., two seconds, the potential differences from each pair of bipolar electrodes were band-pass filtered ranging from 0.5 to 2 Hz to capture major kinematic information of 1 Hz. It was observed that movement peaks happened when a finger was fully flexed, and those peaks were identified using the following criteria. Firstly, the amplitudes at the prospective peaks were at least 200 microvolt (µV). Secondly, these movement peaks occurred 400 milliseconds (ms) after stimulus onsets, since the reaction time from visual stimulus to movement onset was about 180 ms [52] and the time reaching the peak from movement onset was usually longer than 200 ms. Thirdly, movement peaks in the last 500 ms of each trial were not used because their corresponding EEG data might be contaminated by the following trial. Lastly, movement peaks were at least 200 ms apart from each other and, if there were multiple peaks within 400 ms time window, the peak with the maximal strength was selected. Trials in which subjects made wrong movements were removed. Numbers of detected finger movements and their distributions among individual fingers were listed in table 1. Then, EEG data centered at corresponding finger movement peaks in all trials were extracted with the length of one second and categorized into different fingers. For ECoG data, the similar procedure for the detection of finger movements was performed. Position data from the data glove was in the range of [−5, 10] (with arbitrary unit). Finger movement peaks were identified using two criteria: above the threshold of 2 and peaks at least 200 ms apart and the one with maximal strength selected if there are multiple peaks within 400 ms. ECoG data within one second window corresponding to each movement peak was extracted.

#### 2.3. Power spectral analysis

Both EEG and ECoG data were re-referenced using a common average reference (CAR) before the following analysis, which could enhance SNR [53]: 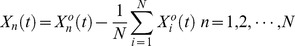
(1)where 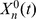
and 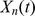 are EEG or ECoG signals on channel *n* and at time *t* before and after CAR, and *N* is the total number of channels.

To calculate power spectral densities (PSDs) of EEG/ECoG on each channel, data from a short-time window *T* centered at movement peaks 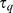 and resting conditions were used, where 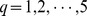 refers to time windows for different fingers, and 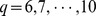 refers to time windows of corresponding resting conditions of different fingers. Resting conditions were defined as one-second length window centered at the middle of two-second fixation windows (i.e., 3 s in Fig. 1(a)). PSDs were then calculated using a windowed Fourier transform [42]: 
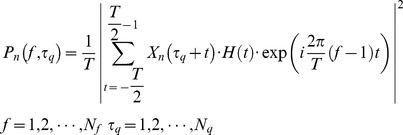
(2)where 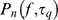 is the PSD at frequency *f* and time 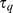 on channel *n*, 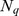 is the number of movements (including corresponding resting conditions). The Hanning window 
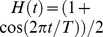
 was used with the window length *T* of one second. The upper-bound frequency 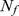 was 125 Hz in EEG data and 200 Hz in ECoG data.

#### 2.4. Principal component analysis

To evaluate movement-related changes in EEG, the principal component analysis (PCA) [43] was applied to PSD data from both movement trials and resting trials in order to identify movement-related spectral structures. In the present study, difference related to changes in movement-related spectral structures was evaluated by comparing a pair of any fingers from one hand. Every time, PSD data from a pair of conditions (i.e., fingers) to be compared and their corresponding resting data were grouped for the PCA analysis using the following procedure, which was repeated for all ten pairs of conditions from five fingers. Grouped PSD data were firstly element-wise normalized to the ensemble average spectrum at each frequency and then the logarithm was taken: 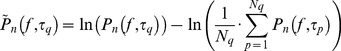
(3)where 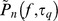 is the log-normalized PSD at frequency *f* and time 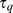 on channel *n*. The purpose of normalization was to evaluate increased or decreased changes in specific spectral structures that would be identified with PCA as discussed below. The purpose of logarithm operation was to treat increased changes (ranging from zero to infinity after logarithm) and decreased changes (ranging from negative infinity to zero after logarithm) equally [42].

The PCA method [43] was then applied to seek the most representative spectral structures in PSD data 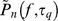, which calculates the eigenvalues 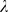 and eigenvectors 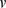 of the covariance matrix 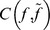 of 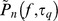 among frequencies 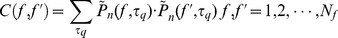
(4)where 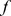 and 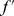 are frequencies and 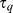 are from the pair of fingers compared. The covariance matrix reveals the correlation between power spectra of every two frequency bins. Its eigenvectors 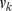 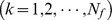 (principal component, PC) define a set of spectral structures in PSD data and their contributions to the variance of PSD data are reflected in corresponding eigenvalues 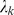. Rearranging PCs according to eigenvalues in a descending order, which forms a set of orthogonal basis in the frequency domain denoted as 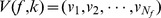. The projection of PSD data from each trial onto the new basis 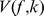 can then be calculated as 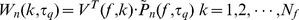
(5)where 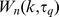 are the weights of PSD on n^th^ channel from movement (and resting) data 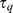 projected onto the k^th^ PC. Projection weights were grouped according to conditions (resting data were also separated according to finger moved after it) and compared to illustrate difference of movement-related changes in spectral structures from different finger movements.

#### 2.5 Feature selection

The feature selection procedure includes the feature extraction using the spectral PCA described in the previous section and the selection of EEG channels that are most discriminative to different fingers. Before these steps, each dataset was separated into training and testing data according to five-fold cross validation. The whole dataset was equally divided into five mutually exclusive subsets, with four subsets (80%) as training data and the rest (20%) for testing. The process was repeated five times and every subset was used for testing once. Thus, EEG features selected for the following classification analysis were obtained from the training data only.

In the present study, the first five PCs were considered as feature PCs. Projection weights from each of these PCs and their combinations were used to decode movement-related changes in EEG spectral structures when different fingers were moved. To achieve optimal classification performance, spatial patterns of projections on spectral structures were further considered. For the spectral data on each channel, the PCA analysis was performed and projection weights of the first five PCs were obtained for each pair of fingers to be compared on the channel. To identify channels with the large differences in projection weights between two different fingers, the r^2^ values were calculated on each channel [54], which evaluates the proportion of variance between two data sets accounted by the difference of their means: 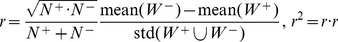
(6)where 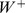 and 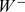 contain projection weights on PCs (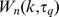 in equation (5)) from two conditions, and 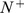
and 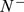 are the numbers of samples in each condition. Channels indicating significant r^2^ values in comparisons were selected as feature channels (Fig. 1(c) and see section 2 in Results), and projection weights on these channels were used as input features for classifiers (discussed in next section) to decode finger movements.

### 3. Classifications of finger movements

Using features discussed in previous section, classifications were performed on 20% testing data to distinguish finger movements in pairs (e.g., thumb vs. index) to investigate the difference in movement-related EEG spectral changes from different fingers. Ten pairs of comparisons were performed, i.e., thumb vs. index, thumb vs. middle, thumb vs. ring, thumb vs. little, index vs. middle, index vs. ring, index vs. little, middle vs. ring, middle vs. little, and ring vs. little. Since the classification of two conditions was a two-class classification problem, a binary classifier was applied, i.e., the linear support vector machine (SVM) method [55], [56] with the radial basis kernel function (RBF) from the LIBSVM package [57]. Briefly, the method maps input feature data into a high dimensional space and seeks an optimal separating hyper plane that has maximal margins between two classes of data samples. The penalty parameter and gamma value in RBF kernel were determined by a grid-search approach [58].

### 4. Evaluation of decoding performance

Decoding accuracy (DA) was defined as the number of correctly classified movements divided by the total number of movements [59]. To get an unbiased estimation of decoding accuracy, EEG/ECoG trial data of individual movements were randomly permuted before going through the five-fold cross validation. The whole process was repeated twenty times, on which the mean and variance of decoding accuracies were calculated.

The significance of achieved classification accuracy was also compared with respect to the empirical guessing level. The empirical guessing level p for each pair of compared fingers was calculated using a permutation test. During the test, the class labels were randomly permuted 500 times and the same classification procedure was performed on obtained dataset in each permutation as on the original dataset. The decoding accuracies from all permutations were then averaged to obtain the empirical guessing level and Student t-test was used to test the significance between comparisons. In a two-class classification problem, the probability (*p*) and its associated confidence intervals were given as [60]

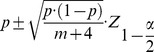
(7) where *m* is the number of total movements from two conditions compared, 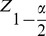 is the 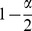 quantile of standard normal distribution, and 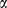 is the significance level, such as 0.05. The decoding performance of a classifier is considered to be statistically significant from the guessing level if it is beyond the confidence intervals of guessing level with a significance level *α*.

To evaluate the decoding performance using the broadband feature from PCA as compared to other spectral features in EEG, spectral powers from individual frequency bands including alpha (8∼12 Hz), beta (13∼30 Hz) and gamma (>40 Hz) in EEG data from each subject were also extracted and applied to evaluate accuracy using the same classification procedure.

## Results

### 1. Principal components in EEG and ECoG data

**Figure 2:**
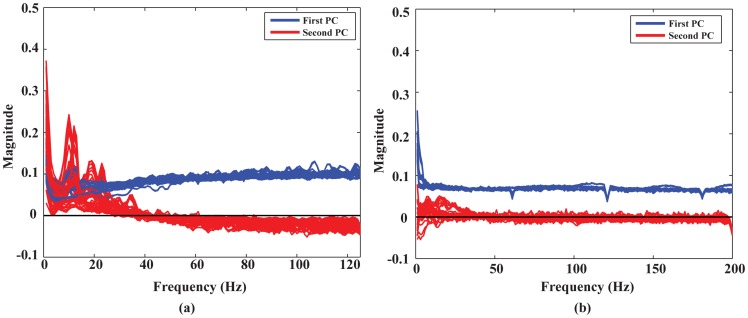
First and second principal components obtained in both EEG and ECoG data from ten pairs of finger movements in all subjects. Each curve is the averaged 1^st^ or 2^nd^ principal component across 50 electrodes (figure 1(c)) from one pair of fingers in one subject. (a) EEG data (1–125 Hz). (b) ECoG data (1–200 Hz).

Figure 2 illustrates the first and second PCs of EEG and ECoG data from all comparisons (i.e., ten) in all subjects (i.e., 10 for EEG and 3 for ECoG) on the axes of frequency. Figure 2(a) shows that, for EEG, the first PCs are of non-zero value (around 0.1) over the whole frequency band (up to 125 Hz), which is consistent over all comparisons of different finger movements, over distributed EEG channels (see next section), and over all subjects. Moreover, these first PCs are of the same signs and closer to each other than they are to zero. The second PCs indicate peaks within the *α* band (8–12 Hz) and *β* band (around 20–25 Hz) while the high frequency component (>40 Hz) is near zero. These phenomena are similar to the results obtained from ECoG data (figure 2(b)), in which the first PCs have the same positively signed magnitudes (around 0.07) over the whole frequency band (up to 200 Hz) over all comparisons of finger pairs and all subjects and the second PCs have elevated deflections away from zero within *α*/*β* frequency bands as well. However, it is worth to note that the first PCs in EEG present a slightly increasing pattern in the low frequency range as compared to the ones in ECoG.

As discussed in the ECoG study [42], the first PC with non-zero magnitudes captures the broadband frequency change during finger movements, while the second PC reflects the power-decreasing rhythms in low frequency bands, consistent with event-related desynchronization (ERD) due to movements [61]. These results indicate that movement-related spectral structures reported in previous ECoG studies [42] are also available and can be identified in EEG data.

### 2. Spatial patterns of movement-related spectral changes

**Figure 3:**
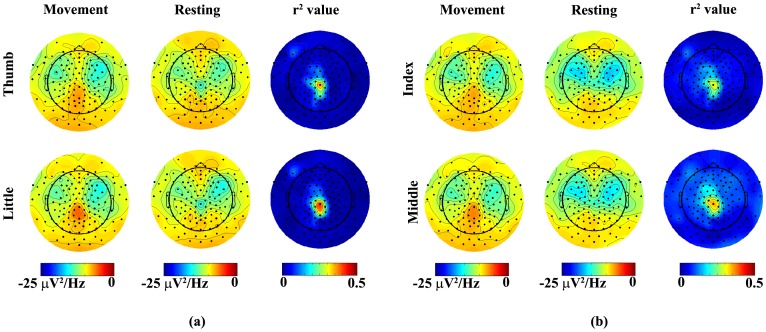
Topographies of project weights on the 1^st^ PC averaged over all subjects in two representative pairs of fingers: (a) thumb vs. little (b) index vs. middle. Left column: projection weights from movement data; Middle column: weights from corresponding resting conditions data prior to movements; Right column: r^2^ value between projection weights from movement and resting data.

Since the first PC with non-zero magnitudes observed in EEG is in concordance with previous ECoG studies, in which the projection weights on this PC are found specific to different finger movements [42], projection weights on the first PC from all channels were studied to understand movement-related spectral changes spatially. Figure 3 illustrates the topographies of projection weights of EEG PSDs upon the first PC in two comparisons (thumb vs. little and index vs. middle). It is observed that large projection weights from both movement conditions and resting conditions appear on both left and right fronto-central areas and gradually decrease toward centro-parietal area around the midline. By comparing the projection weights of PSD data from movements and those from their corresponding resting conditions in each finger (the first two columns of each row in figures 3(a) and (b)), it indicates small magnitude differences in both left and right central areas and large difference over the centro-parietal area that is slightly toward the left side of midline. These observations are further confirmed in the topographies of r^2^ values, which are about 0.2 to 0.25 in the central areas and reach 0.5 over the centro-parietal area (the right column of each row in figures 3(a) and (b)). When spatial patterns of projection weights from different finger movements are compared (thumb vs. little in figure 3(a) and index vs. middle in figure 3(b)), the magnitude differences were observed in the left and right fronto-central and centro-parietal areas while the general patterns are maintained in all fingers. Furthermore, the movement-related spectral changes in the centro-parietal area seem more significant than both left and right fronto-central areas, which is similar to observations when PSD data from movements and resting are compared. On the contrary, spatial patterns of projection weights from resting PSD data that are corresponding to different finger movements do not indicate such difference (middle columns of figures 3(a) and (b)), which suggests that the difference of spatial patterns of projection weights only exists during movements. Based on these results, 50 electrodes covering areas discussed above (figure 1(c)) were selected as feature channels to perform decoding in the following analysis. Other channel sets were also tested to evaluate the effect of channel locations on decoding accuracy (see section 6 in Results).

### 3. Decoding accuracy of movements using EEG and ECoG data

**Figure 4:**
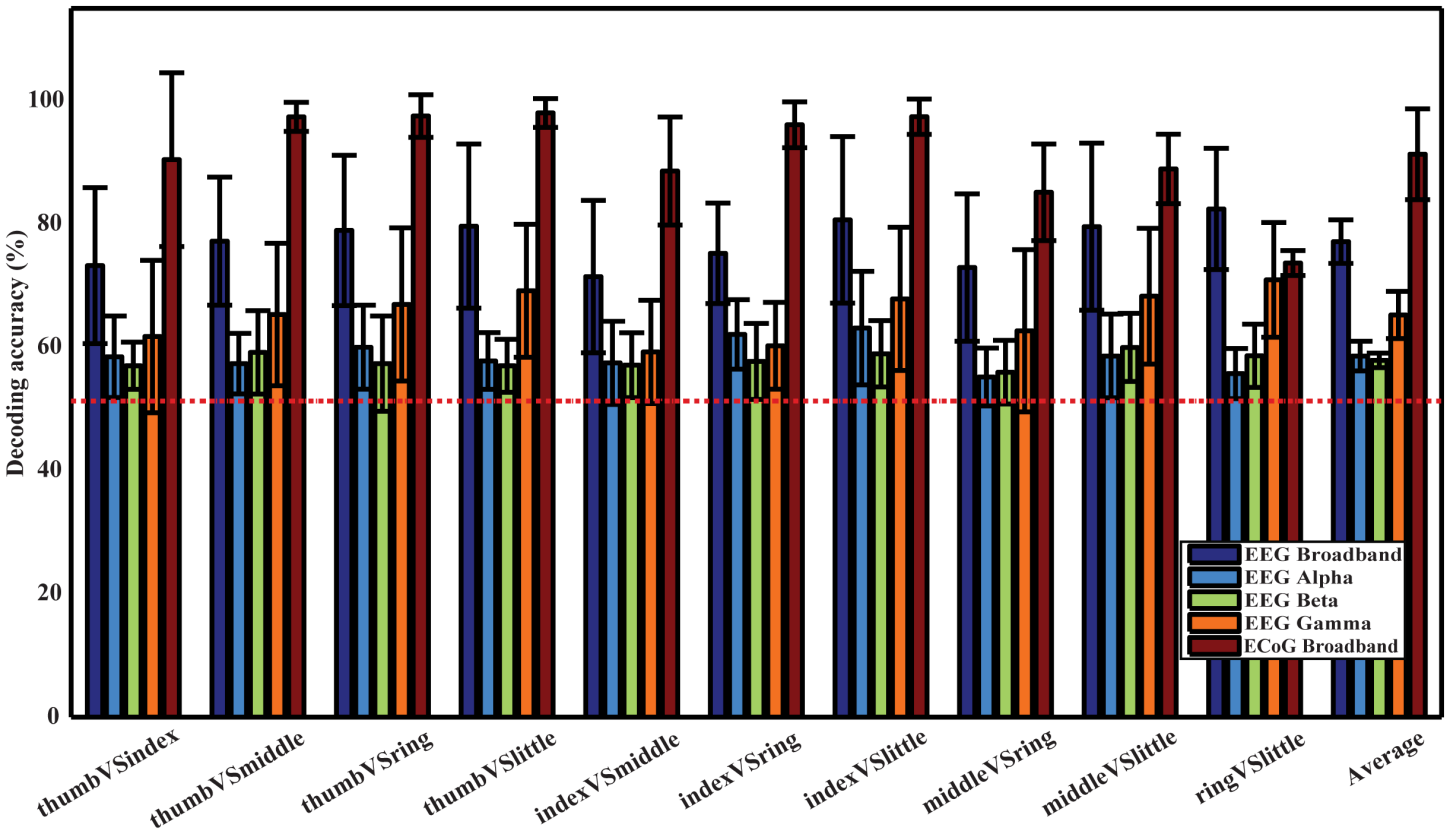
Decoding accuracies for ten pairs of fingers from one hand and their average DAs using EEG and ECoG with the broadband spectral feature from EEG, EEG spectral power in alpha band, EEG spectral power in beta band, EEG spectral power in gamma band, and broadband spectral feature from ECoG. The red dashed line indicates the empirical guessing level of 51.26% and the vertical lines indicate standard deviations.

Figure 4 shows the mean decoding accuracies and corresponding standard deviations using EEG and ECoG, respectively, calculated from 20 permutations and all subjects. It indicates that DAs from ten comparisons using the broadband feature are all higher than 70% for EEG, with the lowest DA of 71.43% in index vs. middle and the highest DA of 82.41% in ring vs. little. The average DA across all pairs of fingers and subjects is 77.11%. ECoG produces better decoding performance, with the lowest DA of 73.64% in ring vs. little and the highest DA of 97.98% in thumb vs. little. The average DA is 91.28% across all finger pairs and subjects. Furthermore, DAs using both EEG and ECoG are significantly higher than the empirical guessing level 51.26% (the red horizontal dashed line) in one-sample t-test (*p*<*0.05*). And the broadband feature from ECoG yields significantly higher DAs than it from EEG (*p*<*0.05*).

The average DAs achieved by spectral features in the three frequency bands are 58.55% (alpha), 57.86% (beta), and 65.21% (gamma), respectively and all reach significant level against the empirical guessing level (*p*<*0.05*). Among these features, gamma band yields significantly higher DAs than the other two (*p*<*0.05*), while the difference between alpha and beta band is not significant (*p*>*0.05*). Furthermore, the broadband feature has significantly higher DAs than the feature from any of these individual frequency bands (*p*<*0.05*).

The significance of decoding performance for each pair of fingers using EEG data is listed in table 2. With the significance level *α* as 0.05, most of decoding accuracies for all subjects and all finger pairs were above the upper bound of 95% confidence interval of guessing, except four pairs out of one hundred (underlined ones in table 2). These results demonstrate that almost all decoding accuracies of ten finger pairs from all subjects are significantly better than the guessing level.

**Table 2:** Significance tests of decoding accuracy for ten pairs of fingers from one hand using EEG data in all subjects (averaged decoding accuracy/upper bound of (1-*α*) confidence level, *α* = 0.05).

	thumb vs. index	thumb vs. middle	thumb vs. ring	thumb vs. little	index vs. middle	index vs. ring	index vs. little	middle vs. ring	middle vs. little	ring vs. little
Subj. 1	68.89/58.15	69.82/57.56	83.21/58.53	93.33/58.32	62.97/56.56	77.03/56.82	94.50/56.59	64.23/56.83	91.87/56.50	91.47/56.57
Subj. 2	67.36/56.55	70.62/60.76	71.11/56.93	63.03/56.12	72.74/58.02	69.95/55.57	78.14/56.56	63.14/57.93	68.23/61.16	71.92/57.14
Subj. 3	75.12/60.20	75.73/57.90	87.57/57.32	76.63/57.76	89.74/63.79	87.22/63.10	80.04/63.29	94.91/55.95	71.19/56.31	90.34/56.74
Subj. 4	66.77/56.92	67.61/56.40	70.03/56.80	82.86/59.95	57.53/57.51	63.84/56.64	92.39/61.72	64.67/56.71	94.13/58.79	86.95/60.43
Subj. 5	63.06/65.03	76.40/60.61	72.00/57.53	78.00/57.88	67.37/58.40	70.86/63.51	76.12/67.24	81.87/58.81	75.86/62.55	70.62/57.96
Subj. 6	56.65/56.49	66.72/56.40	74.32/56.93	95.87/56.27	62.00/56.30	63.40/56.43	95.70/56.62	55.16/56.47	96.97/57.38	97.77/56.99
Subj. 7	71.50/56.61	73.65/56.82	58.20/56.51	75.50/56.30	55.13/56.92	64.67/56.52	57.97/56.52	69.26/56.17	55.71/56.68	72.90/56.50
Subj. 8	56.88/56.29	57.48/56.56	67.58/56.43	59.13/56.68	65.13/56.13	71.29/56.80	58.22/56.15	62.03/56.28	62.32/56.30	67.97/57.11
Subj. 9	99.16/55.40	98.77/56.81	99.09/56.83	94.06/56.51	62.61/56.76	81.25/56.51	83.06/56.68	71.97/57.08	76.39/57.23	70.44/56.51
Subj.10	87.29/56.68	86.56/56.79	92.39/57.12	63.25/56.37	96.29/56.34	85.33/57.00	80.23/56.44	84.23/56.98	86.78/56.45	81.74/57.26
Mean	71.27	74.34	77.55	78.16	69.15	73.48	79.64	71.15	77.94	80.21
Std.	13.25	11.43	12.60	13.51	13.57	8.81	13.32	12.24	14.07	10.78

The last two rows of the table show the means and standard deviations of decoding accuracies of each pair of fingers across all subjects. Classification results were obtained from using the 50-channel set. Values underlined are not significantly higher than the guessing level.

### 4. Decoding using data from resting conditions

**Figure 5:**
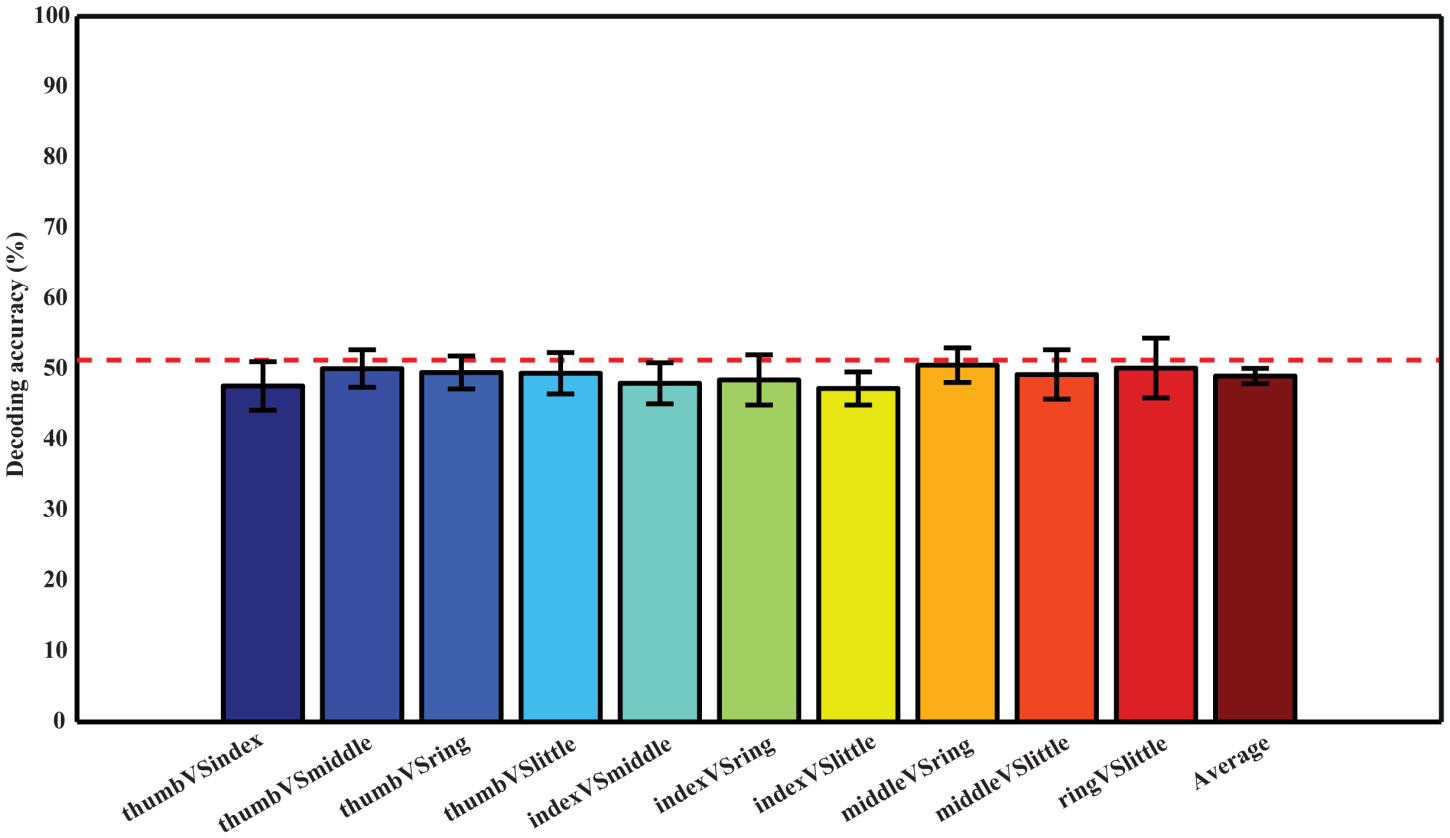
Decoding accuracies using the resting condition EEG data prior to movements in all subjects. Classifications were done using the first three PCs and 50 EEG channels (figure 1(c)). Ten pairs are displayed in the same sequence as in figure 4. The red dashed line shows the empirical guessing level of 51.26%.

To further verify that it was movement-related changes in EEG data that contributed to the decoding accuracies in figure 4, the same classification procedure was performed on data from resting conditions prior to individual finger movements (they were categorized to different fingers according to movements performed after). Figure 5 shows that DAs for all pairs of fingers are at the guessing level, in the range from 47.24% (index vs. little) to 50.55% (middle vs. ring).

### 5. Decoding accuracies using different principal components

**Figure 6:**
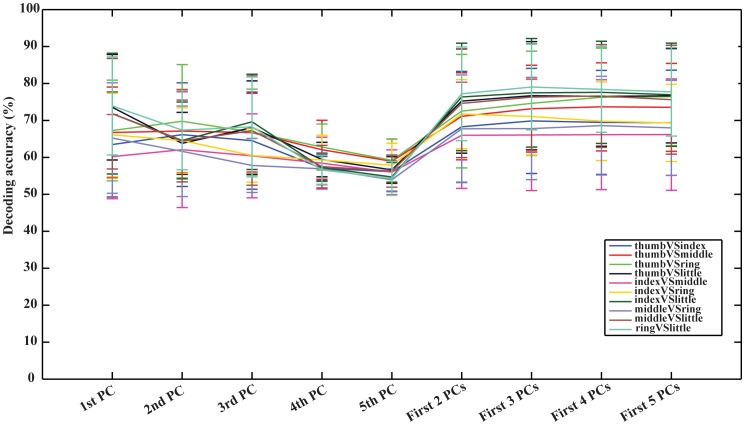
Decoding accuracies using single and multiple principal component(s) in all subjects. Classifications were done using 50 EEG channels (figure 1(c)).

Figure 6 illustrates the average decoding accuracies in ten pairs of fingers from EEG signals using projection weights on single (from first to fifth) or multiple (from first two to first five) PC(s) as input features for classification. As far as single PC is concerned, the first PC produces higher DAs than other single PCs, while the differences are not significant against the second and third PCs (*p* = *0.22* and *0.17*, respectively). In most cases, the decoding accuracy of each pair of fingers decreases from the first PC to the fifth PC, indicating that the spectral structure in the first PC is more relevant to movements performed by fingers than other PCs.

When multiple PCs are concerned, it suggests higher DAs than single PCs with the statistical significance (*p*<*0.05*). The highest DA can usually be achieved when projection weights on first three PCs are used as input features for classification. It further indicates that three pairs of fingers (thumb vs. little, ring vs. little, and thumb vs. index) achieve the highest DAs using the first three PCs, and others have their highest DAs using the first two, four, or five PCs. None of them gets the highest DA from single PCs.

### 6. Decoding accuracy of different channel sets

**Figure 7:**
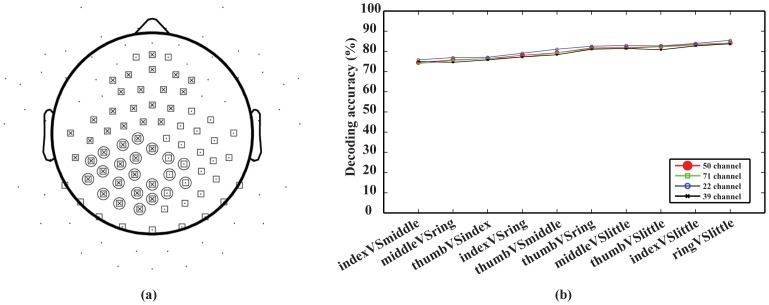
Comparison of decoding accuracies using different numbers of EEG channels. Data were from the optimal principal component set and averaged over all subjects. (a) Layout of three channel sets: 22 channels (circle), 39 channels (cross), and 71 channels (square). A black dot indicates one electrode on the scalp. (b) Decoding accuracy of four channels sets at ten pairs of finger movements.

To evaluate the effect of different feature channels on the decoding performance, four different channel sets (22, 39, 50, and 71 channels) were chosen. Their spatial layouts are illustrated in figure 7(a). The 22-channel set was chosen to cover the centro-parietal area, which indicates the largest difference in maps of projection weights among different movements (figure 3). The 39-channel set covered the area of 22 channels and left fronto-central area since all subjects used right hands to perform the tasks. The 71-channel set included more electrodes on the occipital area than the 50-channel set (figure 1(c)), which covered both left and right centro-parietal areas. The present results indicate that decoding accuracies using different channel sets (figure 7(b)) were close to each other. In the pairwise Student t-test, no significant difference could be identified in terms of performance using different channel sets (*p*>*0.05*).

## Discussion

BCI is an assistive technology, which allows people suffering from severe motor disabilities to control external devices by directly decoding brain signals and bypassing impaired peripheral nerves and muscles [62]. One challenge that impedes the development of noninvasive BCIs is the limited number of available control signals. Recent ECoG studies that demonstrated the feasibility of decoding individual finger movements from one hand using decoupled movement-related spectral changes [42] shed lights on using EEGs to decode individual finger movements, which could greatly increase the degree of freedom of noninvasive BCIs. The aim of the present study is to investigate whether similar movement-related spectral changes can be detected in EEG as in ECoG and its efficacy in discriminate individual finger movements from one hand. Similar findings as in ECoG [42] were observed in the present study in EEG. A spectral structure of non-zero magnitudes with positive signs across the whole frequency band of EEG was identified similarly as in ECoG (figure 2), which is sensitive to movements performed by individual fingers specifically on channels over left and right fronto-central and midline centro-parietal areas in all subjects. A second spectral structure was also identified with spectral peaks within both alpha and beta bands and zero magnitudes in the frequency band over 40 Hz, which resembles the well-known ERD phenomena [61] in low frequency bands. These results confirmed that EEG signals contain movement-related information similar to invasive ECoG signals. These movement-related spectral structures were then utilized to decode individual finger movements from one hand.

An average decoding accuracy of 77.11% was achieved in distinguishing movements performed by all pairs of different fingers from one hand in ten subjects. Decoding performance was stable across different pairs of fingers (std: 3.72%, figure 4). When examining decoding accuracies of each pair of fingers across different subjects, average decoding accuracies were all above the guessing level with the similar level of variations (Table 2). Resting data prior to movements do not contain information to accurately decode movements (figure 5), indicating EEG spectral changes identified in comparisons between different movements and between movements and resting are indeed induced by movements. Although the decoding accuracy using EEG is lower than ECoG (averaged in 91.28% in three epilepsy patients), the DAs of all finger pairs using EEG are significantly higher than the guessing level (*p*<*0.05*), demonstrating the feasibility of using such features in discriminating individual finger movements from one hand. The plausible reason for the reduction in decoding accuracy is that EEG records neural electrical potentials from the scalp, where signals are greatly attenuated during the propagation through tissues, i.e., the brain, skull, and scalp. One more reason that may also reduce the decoding accuracy of EEG is the movement frequency (twice in EEG recordings versus 3 to 5 times in ECoG recordings in the time window of two seconds) and faster movements are expected to elicit stronger signals, which is worthy of further investigations.

Many studies have been conducted to decode movements of different hand parts using noninvasive EEG. Studies to distinguish movements of fingers from wrists and/or hands have been reported [33], [63]–[65]. For example, left and right wrist movement imageries using EEG were classified with DA of 89% [63]. Average DAs from real movement and motor imagery of wrist and finger from same hands using EEG were reported as 71% [33]. DA for left and right hand movements using EEG was 75% [64], and DA for motor imagery was 85% by using Hidden Markov Models as classifier [65]. There are also studies dedicated to distinguishing fingers movement form both hands [66]–[70]. To name a few, single-trial DA for left versus right index finger movements using EEG was around 80% [66]. DA for left or right finger tapping using EEG was 78% [67]. Self-paced key press task of left vs. right index finger was reported with DA of 92.1% [68] and 85% for data from the BCI competition 2003 [69]. Another noninvasive study using MEG to decode left and right index finger movements obtained DA of 80–94% for five subjects [70].

To our knowledge, this is one of the first few studies to decode individual finger movements from one hand using noninvasive EEG. Some studies aimed to decode finger movements from the same hand [71], [72]. One of these studies discussed the decoding of four fingers movements (no ring finger) using MEG and EEG and suggested the EEG recording was not very robust in part due to its low spatial resolution [71]. Another one decoded the index finger against the little finger from the same hand, resulting in decoding accuracies of 53–81% [72]. The decoding accuracy (77.17% in average) achieved in the present study is comparable or even superior to some of those studies, which indicates that robust decoding can be achieved using EEG spectral power changes obtained from the proposed method. Note that this is achieved by decoding all pairs of fingers from one hand, which is much harder than decoding specific fingers (such as the index) from both hands, decoding both hand movements, or decoding fingers from wrists in same hands, because movements of fingers in one hand activate close areas in the sensorimotor cortex within same hemispheres and such activations are much smaller compared to movements of other large body parts, such as wrists or hands [27]. The decoding accuracy using ECoG in the present study (91.28% in average) is also superior to other reported studies using ECoG. For example, the average decoding accuracy using the same dataset in same three subjects was 80.8% [39]. In another study, optimal DAs to distinguish thumb and index movements in one hand were 83.6% and 90% in two subjects [73]. Although not reaching 90%, the general acceptable decoding rate by BCI potential users [74], its improvements to the level for practical uses are expected by improving signal processing methods in feature extraction, classification algorithms, optimal control protocols (such as using a pair of fingers with better and reliable DA), and subject training.

Principal component analysis [43] was performed to decompose EEG PSD data and extract movement-related spectral structures that change when different fingers move. It is worth to note that PCA methods have been applied to study finger and/or hand movements previously [42], [75], [76]. In the present study, principal components in spectral domains were identified by applying PCA on spectra data. The first spectral PC indicates a close-to-uniform spectrum over the whole frequency range, including gamma band (>40 Hz). When comparing with the ones obtained in ECoG, the first spectral PC from EEG presents a slight increasing profile in the low frequency range. Movement-related power changes in high frequency bands, i.e., gamma band, have been found in EEG data recorded during real and/or imagined motor movements [32], [77], [78], and have been applied in BCI problems [63], [79]. Studies based on invasive measurement techniques also validated these spectral power changes [7], [80] and utilized them to discriminate individual finger movements [37]. It has been suggested that the spectral structure indicated in the first principal component might be the extension of gamma band activity to the corresponding low frequency bands [42]. The better decoding data than other principal components (figure 6) further indicate that this principle component is sensitive to movements performed by fingers. The same PC was also similarly detected in ECoG data with the improved DA (91.28%), which is much higher than the DA (80.8%) achieved in a previous study [39] on the same ECoG data with best features from gamma band (65–200 Hz). These facts suggest that this characteristic spectral structure is universally available in electrical potential data (including EEG and ECoG). It is a movement-related spectral feature that is more specific to finger movements than spectral features in alpha and beta bands in the second PC that actually have been well studied and widely used in BCI [6], [24], [81] and is more complete than spectral features in gamma bands [42]. Furthermore, decoding accuracy using features from multiple principal components, e.g., first three PCs, is higher than the first PC, which indicates the contributions from other PCs (such as the second PC). However, adding more than first three PCs does not further increase decoding accuracy (figure 6), which indicates that the PCs corresponding to small eigenvalues are more likely related to noise or other spectral structures rather than movement-related spectral structures.

While it has been suggested that changes on identified movement-related spectral structure caused by the movement of each finger can be localized to a single electrode in ECoG [42], it is not likely in EEG data due to the volume conductor effect [82], [83]. Large differences on the identified movement-related spectral structure between movements and corresponding resting conditions were mainly shown in the centro-parietal area centered on the immediately left side of midline (figure 3). Furthermore, relatively small differences were observed in both left and right fronto-central areas (figure 3). These areas include the primary motor cortex, primary somato-sensory cortex, and fronto-parietal network that are involved in the control and execution of finger movements [84]. The focus on the left side of midline is contralateral to the moving hand (i.e., right hands in all subjects), which is consistent with physiological evidence in hand representations on the human cortex. The comparisons between different finger movements also indicate changes on identified movement-related spectral structure over the similar areas (figure 3). These results are consistent with findings from other studies. For example, in a recent EEG study [5], the inferior parietal lobe and the contralateral primary sensorimotor region are found in encoding hand movement kinematics. Thus, multiple channels (e.g. 22 channels on the left centro-parietal area) over a wide surface area instead of individual EEG channels as feature channels are preferable to decode finger movements in EEG. The investigations using different numbers of channels and different spatial coverage of channels did not indicate obvious difference in decoding performance (figure 7) if they cover areas of difference as discussed above. More channels (e.g., 71 channels) did not necessarily lead to better decoding accuracy, due to the fact that more unrelated information was introduced for classification with more channels.

It is noted that the present study focused on testing the availability of movement-related spectral features in EEG caused by individual finger movements and its potential applications to increase the degree of freedom in EEG-based noninvasive BCIs, which was demonstrated by decoding individual fingers from one hand pairwise. It will be useful if the binary classification problem in the present study can be expanded to a multi-class classification problem, which decodes five individual fingers from one hand simultaneously. While the decoding accuracy is expected to be lower than pairwise comparisons and it poses more difficulties to be used in practical BCI systems as discussed above, such a research will be meaningful to understand movement related brain signal changes corresponding individual fingers and thus may facilitate the design and development of hand prostheses that can be directly controlled by brain signals. For example, the multi-class classification can help investigating the co-activation of individual fingers by examining the confusion matrix of classification accuracies [85]. Furthermore, the same concept can be used to directly decode functional units (rather than individual fingers) that are responsible for performing certain complex hand gestures [86], which are more efficient in controlling hand prostheses to finish a real-world task. The evaluation of such decoding tasks under the current framework is worthy of exploring in the future, which can extend the application of the identified EEG feature in noninvasive BCIs to the application of hand prostheses.

## Conclusion

The present study investigated the discrimination of individual fingers from one hand using noninvasive EEG. The experimental results demonstrated that a movement-related spectral structure could be decoupled from EEG power spectrum density data using principal component analysis, similar to findings in ECoG [42]. The movement-related spectral changes in EEG revealed by the proposed method could be utilized to decode individual finger movements with an average decoding accuracy of 77.11% for all pairs of fingers from one hand in all subjects, significantly better than those from individual frequency bands, such as alpha, beta and gamma bands. These promising results can significantly increase the control dimension of EEG-based noninvasive BCI technologies and potentially facilitate their developments with rich control signals to drive complex applications.

## References

[1] Wolpaw JR, Birbaumer N, McFarland DJ, Pfurtscheller G, Vaughan TM (2002) Brain-computer interfaces for communication and control. Clinical Neurophysiology 113: 767–791. 12048038 10.1016/s1388-2457(02)00057-3

[2] Birbaumer N (2006) Breaking the silence: Brain–computer interfaces (BCI) for communication and motor control. Psychophysiology 43: 517–532. 17076808 10.1111/j.1469-8986.2006.00456.x

[3] Schwartz AB (2004) Cortical neural prosthetics. Annual Review of Neuroscience 27: 487–507. 10.1146/annurev.neuro.27.070203.144233 15217341

[4] Schwartz AB, Cui XT, Weber Douglas J, Moran DW (2006) Brain-Controlled Interfaces: Movement Restoration with Neural Prosthetics. Neuron 52: 205–220. 17015237 10.1016/j.neuron.2006.09.019

[5] Bradberry TJ, Gentili RJ, Contreras-Vidal JL (2010) Reconstructing three-dimensional hand movements from noninvasive electroencephalographic signals. The Journal of Neuroscience 30: 3432–3437. 20203202 10.1523/JNEUROSCI.6107-09.2010 PMC6634107

[6] Wolpaw JR, McFarland DJ (2004) Control of a two-dimensional movement signal by a noninvasive brain-computer interface in humans. PNAS 101: 17849–17854. 15585584 10.1073/pnas.0403504101 PMC535103

[7] Miller KJ, Schalk G, Fetz EE, Nijs Md, Ojemann JG, et al (2010) Cortical activity during motor execution, motor imagery, and imagery-based online feedback. PNAS 107: 4430–4435. 20160084 10.1073/pnas.0913697107 PMC2840149

[8] Pistohl T, Schulze-Bonhage A, Aertsen A, Mehring C, Ball T (2012) Decoding natural grasp types from human ECoG. NeuroImage 59: 248–260. 21763434 10.1016/j.neuroimage.2011.06.084

[9] Chang G-C, Kang W-J, Luh J-J, Cheng C-K, Lai J-S, et al (1996) Real-time implementation of electromyogram pattern recognition as a control command of man-machine interface. Medical Engineering and Physics 18: 529–537. 8892237 10.1016/1350-4533(96)00006-9

[10] Boostani R, Moradi MH (2003) Evaluation of the forearm EMG signal features for the control of a prosthetic hand. Physiological Measurement 24: 309–319. 12812417 10.1088/0967-3334/24/2/307

[11] Sitaram R, Caria A, Veit R, Gaber T, Rota G, et al (2007) fMRI brain-computer interface: a tool for neuroscientific research and treatment. Computational Intelligence and Neuroscience 2007: 1–10. 10.1155/2007/25487 PMC2233807 18274615

[12] Yoo SS, Fairneny T, Chen NK, Choo SE, Panych LP, et al (2004) Brain-computer interface using fMRI: spatial navigation by thoughts. Neuroreport 15: 1591–1595. 15232289 10.1097/01.wnr.0000133296.39160.fe

[13] Bradberry TJ, Rong F, Contreras-Vidal JL (2009) Decoding center-out hand velocity from MEG signals during visuomotor adaptation. NeuroImage 47: 1691–1700. 19539036 10.1016/j.neuroimage.2009.06.023

[14] Coyle SM, Ward TE, Markham CM (2007) Brain-computer interface using a simplified functional near-infrared spectroscopy system. Journal of Neural Engineering 4: 219–226. 17873424 10.1088/1741-2560/4/3/007

[15] Wilson JA, Felton EA, Garell PC, Schalk G, Williams JC (2006) ECoG factors underlying multimodal control of a brain-computer interface. IEEE Transactions on Neural Systems and Rehabilitation Engineering 14: 246–250. 16792305 10.1109/TNSRE.2006.875570

[16] Schalk G, Kubánek J, Miller KJ, Anderson NR, Leuthardt EC, et al (2007) Decoding two-dimensional movement trajectories using electrocorticographic signals in humans. Journal of Neural Engineering 4: 264–275. 17873429 10.1088/1741-2560/4/3/012

[17] Lal TN, Hinterberger T, Widman G, Schroeder M, Hill J, et al (2005) Methods towards invasive human brain computer interfaces. Advances in Neural Information Processing System 17: 737–744.

[18] Farwell LA, Donchin E (1988) Talking off the top of your head: toward a mental prothesis utilizing event-related brain potentials. Electroenceph Clin Neurophysiol 70: 510–523. 2461285 10.1016/0013-4694(88)90149-6

[19] Bin G, Gao X, Yan Z, Hong B, Gao S (2009) An online multi-channel SSVEP-based brain-computer interface using a canonical correlation analysis method. Journal of Neural Engineering 6: 046002. 19494422 10.1088/1741-2560/6/4/046002

[20] Gu Y, Dremstrup K, Farina D (2009) Single-trial discrimination of type and speed of wrist movements from EEG recordings. Clinical Neurophysiology 120: 1596–1600. 19535289 10.1016/j.clinph.2009.05.006

[21] Doud AJ, Lucas JP, Pisansky MT, He B (2011) Continuous three-dimensional control of a airtual helicopter using a motor imagery based brain-computer interface. PLoS ONE 6: e26322. 22046274 10.1371/journal.pone.0026322 PMC3202533

[22] Zhou J, Yao J, Deng J, Dewald JPA (2009) EEG-based classification for elbow versus shoulder torque intentions involving stroke subjects. Computers in Biology and Medicine 39: 443–452. 19380125 10.1016/j.compbiomed.2009.02.004 PMC2865155

[23] Pfurtscheller G, Brunner C, Schlögl A, Lopes da Silva FH (2006) Mu rhythm (de)synchronization and EEG single-trial classification of different motor imagery tasks. NeuroImage 31: 153–159. 16443377 10.1016/j.neuroimage.2005.12.003

[24] Morash V, Bai O, Furlani S, Lin P, Hallett M (2008) Classifying EEG signals preceding right hand, left hand, tongue, and right foot movements and motor imageries. Clinical Neurophysiology 119: 2570–2578. 18845473 10.1016/j.clinph.2008.08.013 PMC2602863

[25] Nicolas-Alonso LF, Gomez-Gil J (2012) Brain Computer Interfaces, a Review. Sensors 12: 1211–1279. 22438708 10.3390/s120201211 PMC3304110

[26] Hochberg LR, Donoghue JP (2006) Sensors for brain-computer interfaces. IEEE Engineering in Medicine and Biology Magazine 25: 32–38. 10.1109/memb.2006.1705745 17020197

[27] Pfurtscheller G, Lopes da Silva FH (1999) Event-related EEG/MEG synchronization and desynchronization: basic principles. Clinical Neurophysiology 110: 1842–1857. 10576479 10.1016/s1388-2457(99)00141-8

[28] Nunez PL, Srinivasan R, editors (2006) Electric Fields of the Brain: The Neurophysics of EEG. New York: Oxford University Press.

[29] Acharya S, Fifer MS, Benz HL, Crone NE, Thakor NV (2010) Electrocorticographic amplitude predicts finger positions during slow grasping motions of the hand. Journal of Neural Engineering 7: 046002. 20489239 10.1088/1741-2560/7/4/046002 PMC4021582

[30] Lebedev MA, Nicolelis MAL (2006) Brain-machine interfaces: past, present and future. Trends in Neurosciences 29: 536–546. 16859758 10.1016/j.tins.2006.07.004

[31] Vuckovic A (2009) Non-invasive BCI: How far can we get with motor imagination? Clinical Neurophysiology 120: 1422–1423. 19616995 10.1016/j.clinph.2009.06.007

[32] Waldert S, Preissl H, Demandt E, Braun C, Birbaumer N, et al (2008) Hand movement direction decoded from MEG and EEG. The Journal of Neuroscience 28: 1000–1008. 18216207 10.1523/JNEUROSCI.5171-07.2008 PMC6671004

[33] Mohamed AK, Marwala T, John LR (2011) Single-trial EEG discrimination between wrist and finger movement imagery and execution in a sensorimotor BCI. 2011 Annual International Conference of the IEEE Engineering in Medicine and Biology Society. pp. 6289–6293. 10.1109/IEMBS.2011.6091552 22255776

[34] Zanos S, Miller KJ, Ojemann JG (2008) Electrocorticographic spectral changes associated with ipsilateral individual finger and whole hand movement. 30th Annual International Conference of the IEEE Engineering in Medicine and Biology Society. pp. 5939–5942. 10.1109/IEMBS.2008.4650569 19164072

[35] Flamary R, Rakotomamonjy A (2012) Decoding finger movements from ECoG signals using switching linear models. Frontiers in Neuroscience 6. 10.3389/fnins.2012.00029 PMC3294271 22408601

[36] Liang N, Bougrain L (2012) Decoding Finger Flexion From Band-specific ECoG Signals in Humans. Frontiers in Neuroscience 6. 10.3389/fnins.2012.00091 PMC3384842 22754496

[37] Kubánek J, Miller KJ, Ojemann JG, Wolpaw JR, Schalk G (2009) Decoding flexion of individual fingers using electrocorticographic signals in humans. Journal of Neural Engineering 6: 66001. 10.1088/1741-2560/6/6/066001 PMC3664231 19794237

[38] Shenoy P, Miller KJ, Ojemann JG, Rao RPN (2007) Finger Movement Classification for an Electrocorticographic BCI. 3rd International IEEE/EMBS Conference on Neural Engineering. pp. 192–195.

[39] Onaran I, Ince NF, Cetin AE (2011) Classification of multichannel ECoG related to individual finger movements with redundant spatial projections. Annual International Conference of the IEEE Engineering in Medicine and Biology Society. pp. 5424–5427. 10.1109/IEMBS.2011.6091341 22255564

[40] Samiee S, Hajipour S, Shamsollahi MB (2010) Five-class finger flexion classification using ECoG signals. International Conference on Intelligent and Advanced Systems. pp. 1–4.

[41] Wang W, Degenhart AD, Collinger JL, Vinjamuri R, Sudre GP, et al. (2009) Human motor cortical activity recorded with Micro-ECoG electrodes, during individual finger movements. Annual International Conference of the IEEE Engineering in Medicine and Biology Society pp. 586–589. 10.1109/IEMBS.2009.5333704 PMC3142578 19964229

[42] Miller KJ, Zanos S, Fetz EE, Nijs Md, Ojemann JG (2009) Decoupling the cortical power spectrum reveals real-time representation of individual finger movements in humans. The Journal of Neuroscience 29: 3132–3137. 19279250 10.1523/JNEUROSCI.5506-08.2009 PMC6666461

[43] Glaser EM, Ruchkin DS (1976) Principles of Neurobiological Signal Analysis. New York: Academic Press.

[44] Jain RK, Datta S, Majumder S (2012) Design and control of an EMG driven IPMC based artificial muscle finger. In: Naik GR, editor. Computational Intelligence in Electromyography Analysis - A Perspective on Current Applications and Future Challenges: InTech.

[45] Bundhoo V, Park EJ (2005) Design of an artificial muscle actuated finger towards biomimetic prosthetic hands. Proceedings of 12th International Conference on Advanced Robotics: 368–375.

[46] Miller KJ, Schalk G (2008) Prediction of finger flexion: 4th brain-computer interface data competition. BCI Competition IV.

[47] Schalk G, McFarland DJ, Hinterberger T, Birbaumer N, Wolpaw JR (2004) BCI2000: a general-purpose brain-computer interface (BCI) system. IEEE Transactions on Biomedical Engineering 51: 1034–1043. 15188875 10.1109/TBME.2004.827072

[48] Delorme A, Makeig S (2004) EEGLAB: an open source toolbox for analysis of single-trial EEG dynamics including independent component analysis. Journal of Neuroscience Methods 134: 9–21. 15102499 10.1016/j.jneumeth.2003.10.009

[49] Hyvärinen A, Karhunen J, Oja E (2001) Independent Component Analysis. New York: Wiley.

[50] Bell AJ, Sejnowski TJ (1995) An information-maximization approach to blind separation and blind deconvolution. Neural Computation 7: 1129–1159. 7584893 10.1162/neco.1995.7.6.1129

[51] Mognon A, Jovicich J, Bruzzone L, Buiatti M (2011) ADJUST: An automatic EEG artifact detector based on the joint use of spatial and temporal features. Psychophysiology 48: 229–240. 20636297 10.1111/j.1469-8986.2010.01061.x

[52] Welford AT (1980) Reaction Times. New York: Academic Press.

[53] McFarland DJ, McCane LM, David SV, Wolpaw JR (1997) Spatial filter selection for EEG-based communication. Electroencephalography and Clinical Neurophysiology 103: 386–394. 9305287 10.1016/s0013-4694(97)00022-2

[54] Müller K-R, Krauledat M, Dornhege G, Curio G, Blankertz B (2004) Machine learning techniques for brain-computer interfaces. Biomedical Engineering 49: 11–22. 17271309 10.1109/IEMBS.2004.1404253

[55] Vapnik VN (1998) Statistical Learning Theory. New York: Wiley-Interscience.

[56] Vapnik VN (1999) The Nature of Statistical Learning Theory. New York: Springer.

[57] Chang C-C, Lin C-J (2011) LIBSVM: A library for support vector machines. ACM Transactions on Intelligent Systems and Technology 2: 1–27.

[58] Hsu C-W, Chang C-C, Lin C-J (2010) A practical guide to support vector classication. National Taiwan University.

[59] Han J, Kamber M, Pei J (2012) Data Mining: Concepts and Techniques. MA, USA: Morgan Kaufmann.

[60] Müller-Putz GR, Scherer R, Brunner C, Leeb R, Pfurtscheller G (2008) Better than random: a closer look on BCI results. International Journal of Bioelectromagnetism 10: 52–55.

[61] Pfurtscheller G, Aranibar A (1977) Event-related cortical desynchronization detected by power measurements of scalp EEG. Electroencephalography and Clinical Neurophysiology 42: 817–826. 67933 10.1016/0013-4694(77)90235-8

[62] Wolpaw JR, Birbaumer N, Heetderks WJ, McFarland DJ, Peckham PH, et al (2000) Brain-computer interface technology: a review of the first international meeting. IEEE Transactions on Rehabilitation Engineering 8: 164–173. 10896178 10.1109/tre.2000.847807

[63] Khan YU, Sepulveda F (2010) Brain-computer interface for single-trial eeg classification for wrist movement imagery using spatial filtering in the gamma band. IET Signal Processing 4: 510–517.

[64] Bai O, Lin P, Vorbach S, Li J, Furlani S, et al (2007) Exploration of computational methods for classification of movement intention during human voluntary movement from single trial EEG. Clinical Neurophysiology 118: 2637–2655. 17967559 10.1016/j.clinph.2007.08.025 PMC4154235

[65] Obermaier B, Guger C, Neuper C, Pfurtscheller G (2001) Hidden Markov models for online classification of single trial EEG data. Pattern Recognition Letters 22: 1299–1309.

[66] Lehtonen J, Jylanki P, Kauhanen L, Sams M (2008) Online Classification of Single EEG Trials During Finger Movements. IEEE Transactions on Biomedical Engineering 55: 713–720. 18270008 10.1109/TBME.2007.912653

[67] Liyanage SR, Xu JX, Guan C, Ang KK, Zhang CS, et al. Classification of self-paced finger movements with EEG signals using neural network and evolutionary approaches 2009 9-11 Dec. 20091807–1812.

[68] Yong L, Xiaorong G, Hesheng L, Shangkai G (2004) Classification of single-trial electroencephalogram during finger movement. IEEE Transactions on Biomedical Engineering 51: 1019–1025. 15188873 10.1109/TBME.2004.826688

[69] Xiang L, Dezhong Y, Wu D, Chaoyi L (2007) Combining Spatial Filters for the Classification of Single-Trial EEG in a Finger Movement Task. IEEE Transactions on Biomedical Engineering 54: 821–831. 17518278 10.1109/TBME.2006.889206

[70] Kauhanen L, Nykopp T, Sams M (2006) Classification of single MEG trials related to left and right index finger movements. Clinical Neurophysiology 117: 430–439. 16413826 10.1016/j.clinph.2005.10.024

[71] Quandt F, Reichert C, Hinrichs H, Heinze HJ, Knight RT, et al (2012) Single trial discrimination of individual finger movements on one hand: A combined MEG and EEG study. Neuroimage 59: 3316–3324. 22155040 10.1016/j.neuroimage.2011.11.053 PMC4028834

[72] Blankertz B, Dornhege G, Krauledat M, Muller KR, Kunzmann V, et al (2006) The Berlin brain-computer interface: EEG-based communication without subject training. IEEE Transactions on Neural Systems and Rehabilitation Engineering 14: 147–152. 16792281 10.1109/TNSRE.2006.875557

[73] Scherer R, Zanos SP, Miller KJ, Rao RPN, Ojemann JG (2009) Classification of contralateral and ipsilateral finger movements for electrocorticographic brain-computer interfaces. Neurosurgical Focus 27: E12. 10.3171/2009.4.FOCUS0981 19569887

[74] Huggins J, Wren P, Gruis K (2011) What would brain-computer interface users want? Opinions and priorities of potential users with amyotrophic lateral sclerosis. Amyotroph Lateral Scler 12: 318–324. 21534845 10.3109/17482968.2011.572978 PMC3286341

[75] Soechting JF, Flanders M (1997) Flexibility and Repeatability of Finger Movements During Typing: Analysis of Multiple Degrees of Freedom. Journal of Computational Neuroscience 4: 29–46. 9046450 10.1023/a:1008812426305

[76] Santello M, Soechting JF (1997) Matching object size by controlling finger span and hand shape. Somatosensory and Motor Research 14: 203–212. 9402650 10.1080/08990229771060

[77] Darvas F, Scherer R, Ojemann JG, Rao RP, Miller KJ, et al (2010) High gamma mapping using EEG. NeuroImage 49: 930–938. 19715762 10.1016/j.neuroimage.2009.08.041 PMC2764819

[78] Ball T, Demandt E, Mutschler I, Neitzel E, Mehring C, et al (2008) Movement related activity in the high gamma range of the human EEG. NeuroImage 41: 302–310. 18424182 10.1016/j.neuroimage.2008.02.032

[79] Gonzalez SL, Grave de Peralta R, Thut G, Millán JdR, Morier P, et al (2006) Very high frequency oscillations (VHFO) as a predictor of movement intentions. NeuroImage 32: 170–179. 16631386 10.1016/j.neuroimage.2006.02.041

[80] Shenoy P, Miller KJ, Ojemann JG, Rao RPN (2008) Generalized features for electrocorticographic BCIs. IEEE Transactions on Biomedical Engineering 55: 273–280. 18232371 10.1109/TBME.2007.903528

[81] Huang D, Lin P, Fei D-Y, Chen X, Bai O (2009) Decoding human motor activity from EEG single trials for a discrete two-dimensional cursor control. Journal of Neural Engineering 6: 046005. 19556679 10.1088/1741-2560/6/4/046005

[82] van den Broek SP, Reinders F, Donderwinkel M, Peters MJ (1998) Volume conduction effects in EEG and MEG. Electroencephalography and Clinical Neurophysiology 106: 522–534. 9741752 10.1016/s0013-4694(97)00147-8

[83] Makeig S, Kothe C, Mullen T, Bigdely-Shamlo N, Zhang Z, et al (2012) Evolving Signal Processing for Brain-Computer Interfaces. Proceedings of the IEEE 100: 1567–1584.

[84] Ding L, Ni Y, Sweeney J, He B (2011) Sparse cortical current density imaging in motor potentials induced by finger movement. Journal of Neural Engineering 8: 036008. 21478573 10.1088/1741-2560/8/3/036008 PMC3142475

[85] Dornhege G, del R. Millán J, Hinterberger T, McFarland D, Müller K (2007) Toward Brain-Computer Interfacing. MA, USA: MIT Press.

[86] Ingram JN, Körding KP, Howard IS, Wolpert DM (2008) The statistics of natural hand movements. Exp Brain Res 188: 223–236. 18369608 10.1007/s00221-008-1355-3 PMC2636901

